# The zinc-finger transcription factor MAZR regulates iNKT cell subset differentiation

**DOI:** 10.1007/s00018-019-03119-z

**Published:** 2019-05-07

**Authors:** Maria Jonah Orola, Caroline Tizian, Ci Zhu, Liisa Andersen, Alexandra Franziska Gülich, Marlis Alteneder, Tatjana Stojakovic, Ursula Wiedermann, Michael Trauner, Wilfried Ellmeier, Shinya Sakaguchi

**Affiliations:** 1 Division of Immunobiology, Institute of Immunology, Center for Pathophysiology, Infectiology and Immunology, Medical University of Vienna, 1090 Vienna, Austria; 2 Hans Popper Laboratory of Molecular Hepatology, Division of Gastroenterology and Hepatology, Department of Internal Medicine III, Medical University of Vienna, 1090 Vienna, Austria; 3 Clinical Institute of Medical and Chemical Laboratory Diagnostics, University Hospital Graz, 8036 Graz, Austria; 4 Institute of Specific Prophylaxis and Tropical Medicine, Center for Pathophysiology, Infectiology and Immunology, Medical University of Vienna, 1090 Vienna, Austria; 5 Present Address: Institute of Specific Prophylaxis and Tropical Medicine, Center for Pathophysiology, Infectiology and Immunology, Medical University of Vienna, 1090 Vienna, Austria; 6 Present Address: Institute of Microbiology and Infectious Diseases and Immunology, Charité-University Medical Centre Berlin (CBF), 12203 Berlin, Germany

**Keywords:** iNKT cell subset differentiation, iNKT cell function, Transcriptional regulation, PATZ1/MAZR

## Abstract

Invariant natural killer T (iNKT) cells represent a subgroup of innate-like T cells and play an important role in immune responses against certain pathogens. In addition, they have been linked to autoimmunity and antitumor immunity. iNKT cells consist of several subsets with distinct functions; however, the transcriptional networks controlling iNKT subset differentiation are still not fully characterized. Myc-associated zinc-finger-related factor (MAZR, also known as PATZ1) is an essential transcription factor for CD8^+^ lineage differentiation of conventional T cells. Here, we show that MAZR plays an important role in iNKT cells. T-cell lineage-specific deletion of MAZR resulted in an iNKT cell-intrinsic defect that led to an increase in iNKT2 cell numbers, concurrent with a reduction in iNKT1 and iNKT17 cells. Consistent with the alteration in the subset distribution, deletion of MAZR also resulted in an increase in the percentage of IL-4-producing cells. Moreover, MAZR-deficient iNKT cells displayed an enhanced expression of Erg2 and ThPOK, key factors for iNKT cell generation and subset differentiation, indicating that MAZR controls iNKT cell development through fine-tuning of their expression levels. Taken together, our study identified MAZR as an essential transcription factor regulating iNKT cell subset differentiation and effector function.

**Electronic supplementary material:**

The online version of this article (10.1007/s00018-019-03119-z) contains supplementary material, which is available to authorized users.

## Introduction

Invariant natural killer T (iNKT) cells are a small subgroup of T cells expressing semi-invariant T-cell receptors (TCRs), which consist of an invariant α chain and a limited repertoire of β chains [1, 2]. They recognize lipid antigens such as alpha-galactosylceramide (α-GalCer) presented by CD1d, a non-classical MHC class I molecule, and are capable of rapidly producing a large amount of cytokines upon activation [3, 4]. iNKT cells diverge from conventional T cells at the double-positive (DP) stage of thymocyte development, where iNKT TCR-expressing thymocytes are positively selected by glycolipid–CD1d complexes through DP–DP thymocyte interaction [1, 5, 6]. Upon positive selection, iNKT cell precursors start to express the transcription factor early growth response protein 2 (Egr2) at a high level [7]. This leads to the induction of promyelocytic leukemia zinc finger (PLZF) and c-myc expression, which is accompanied by the acquisition of effector properties and a robust proliferation [8–11]. The classical view (“linear differentiation” model) on iNKT cell differentiation defines, based on the expression of CD24, CD44, CD69 and NK1.1, several sequential iNKT cell maturation stages, starting from the most immature NKT cells designated as stage 0 (CD24^+^CD44^−^CD69^+^), followed by stage 1 (CD24^−^CD44^−^NK1.1^−^), stage 2 (CD24^−^CD44^+^NK1.1^−^) and stage 3 (CD24^−^CD44^+^NK1.1^+^) subsets [6, 12–14]. However, recent studies revealed that each of the classical developmental stages contains terminally differentiated iNKT cells [15, 16]. Based on the expression pattern of the transcription factors PLZF, T-box 21 (T-bet) and RAR-related orphan receptor gamma (RORγt), three distinct iNKT cell subsets have been defined (“lineage diversification” model), analogous to helper T and innate lymphoid cells: iNKT1 (T-bet^hi^PLZF^lo^), iNKT2 (PLZF^hi^RORγt^–^) and iNKT17 (PLZF^mid^RORγt^+^) [6, 17, 18]. iNKT1 cells, which are almost identical to stage 3 iNKT cells, produce both IFN-γ and IL-4 upon activation, whereas the iNKT17 cell subset is included in stage 2 iNKT cells and mainly produces IL-17. iNKT2 cells display a stage 1/stage 2 phenotype and express IL-4. Owing to their unique features, iNKT cells are implicated in controlling a diversity of different types of immune reactions, including antimicrobial responses as well as antitumor immunity [3, 19, 20]. Although key transcription factors controlling iNKT cell development have been identified [5, 14, 17], the fine-tuning of iNKT subset differentiation is not fully understood.

Myc-associated zinc-finger-related factor (MAZR; also known as PATZ1) is a member of the BTB/POZ domain containing zinc-finger transcription factor protein family, which also includes PLZF and T-helper-inducing POZ/Krueppel-like factor (ThPOK; also known as ZBTB7b) proteins [21, 22]. We previously demonstrated that MAZR is a negative regulator of *Cd8* expression in double-negative (DN) thymocytes and that it is part of the transcription factor network controlling helper versus cytotoxic lineage decision of DP thymocytes [23, 24]. MAZR represses ThPOK expression in MHC class I-signaled thymocytes, presumably via binding to the *Thpok* silencer, and thereby prevents the redirection of MHC class I-signaled thymocytes into the helper lineage [24]. More recently, we have also shown that MAZR and Runt-related transcription factor (Runx) proteins synergistically repress ThPOK expression during cytotoxic lineage development and that MAZR is required for the maintenance of ThPOK repression in CD8^+^ T cells [25]. Although these studies revealed an essential role for MAZR at multiple stages of conventional T-cell development, its role in the development of innate-like T cells including iNKT cells remains unknown. MAZR is expressed in the iNKT cell lineage, as reported in the Immunological Genome Project database [26], and a recent transcriptome comparison of each iNKT subset revealed an upregulation of MAZR expression in iNKT2 cells compared to other iNKT cell subsets [27]. Moreover, both ThPOK (which is a MAZR target gene) and Runx proteins (which are MAZR interacting factors) are key regulators of iNKT cell development [28–33]. Together, these data suggest a role for MAZR in iNKT cells. In this study, by analyzing mice with a T-cell-specific deletion of MAZR, we observed an enlargement of the CD44^+^NK1.1^−^ stage 2 iNKT cell population, which was accompanied with elevated expression of CD4 and ThPOK in iNKT cells. The analysis of T-bet, PLZF and RORγt expression revealed that the deletion of MAZR led to an increase in the number of iNKT2 cells, while iNKT1 and iNKT17 cell numbers were reduced in the absence of MAZR. The alteration in iNKT cell subset differentiation, which was caused by iNKT cell-intrinsic defects, resulted in enhanced production of IL-4, along with a reduction in IL-17A secretion, both upon in vitro PMA/ionomycin and in vivo α-GalCer stimulation. Finally, the deletion of MAZR led to an increase in Egr2 expression, a key factor required for the acquisition of an iNKT cell effector program and for iNKT cell subset differentiation [7, 34], at stages 2 and 3 of iNKT cell development. This suggests that MAZR controls iNKT cell development through regulating Egr2 expression, in addition to its repression of ThPOK expression. Together, our data identified MAZR as an essential regulator of iNKT cell subset differentiation.

## Materials and methods

### Mice

*Mazr*^f/f^ [35], *Runx3*^f/f^ [36] and *Thpok*-GFP knock-in [37] mice have been described previously. *Lck*-Cre and *Cd4*-Cre mice [38] were kindly provided by Dr. Chris Wilson. Six-to-ten-week-old mice were used for experiments. All the mice used for experiments were backcrossed onto a C57BL/6 background and were maintained in the preclinical research facility of the Medical University of Vienna. Animal husbandry and experimentation was performed under the national laws (Federal Ministry of Education, Science and Research, Vienna, Austria) and ethics committees of the Medical University of Vienna and according to the guidelines of FELASA, which match that of ARRIVE.

### Cell preparation

Single-cell suspensions of thymocytes and splenocytes were prepared as previously described [23]. For the isolation of hepatic mononuclear cells, livers were perfused with 1 × PBS, were cut into small pieces with scissors and scalpels, and were digested with 600 U/ml collagenase D (Roche) and 10 U/ml DNase I (Roche) in RPMI1640 medium (Sigma) containing 2% FCS (Sigma) for 30 min at 37 °C in a shaker. Subsequently, hepatic cells were filtered through a 70 μm strainer and resuspended in a 35% Percoll (GE Healthcare) solution. After centrifugation at 700*g* at room temperature for 15 min, cells at the high-density fraction were collected and red blood cells were lysed in 1 × BD Pharm buffer (BD Biosciences). Subsequently, cells were stained with appropriate antibodies.

### Enrichment of iNKT cells

For some experiments, thymic and splenic iNKT cells were enriched by negative depletion. Single-cell suspensions of thymocytes and splenocytes were incubated with biotinylated anti-CD8α (53-6.7) and anti-CD19 (6D5) antibodies (Biolegend), followed by the incubation with BD iMag Streptavidin Particles Plus (BD Biosciences). Negative depletion was performed according to the manufacturer’s instruction.

### Antibodies and flow cytometry

Antibodies used in this study are listed in Table S1. Brilliant violet 421-, or PE-conjugated murine CD1d tetramers loaded with PBS-57 (CD1d-tet) were kindly provided by the National Institutes of Health (NIH) Tetramer Facility. Thymocytes, splenocytes, and hepatic monocellular cells were first incubated with eF506 Viability Dye (Thermo Fisher Scientific) as well as purified anti-CD16/CD32 antibody (BD Biosciences) to avoid unspecific antibody binding. Subsequently, appropriate antibodies including CD1d-tet were added and cells were incubated on ice for 30 min. For the intracellular staining of transcription factors, Foxp3/Transcription Factor Staining Buffer Kit (Thermo Fisher Scientific) was used according to manufacture’s instruction. Intracellular MAZR expression was detected by anti-MAZR/PATZ1 antibody (D-5: Santa Cruz Biotechnology), followed by the staining of Alexa Fluor 647 anti-mouse IgG_1_ antibody (RMG1-1: Biolegend). Annexin V staining was performed using Annexin V Apoptosis Detection Kit (Thermo Fisher Scientific). Flow cytometric data were collected with LSRII or Fortessa (BD Biosciences) and were analyzed with Flowjo software (Treestar).

### In vivo BrdU incorporation

Mice were given a single i.p. injection of 1 mg BrdU (Sigma) diluted in 200 μl of 1 × PBS, followed by 2 days BrdU administration in drinking water (1 mg/ml). Mice were then euthanized and single-cell suspensions of thymocytes and splenocytes were prepared. BrdU incorporation was measured using BrdU Flow Kit (BD Biosciences) according to manufacturer’s instruction.

### Measurement of intracellular cytokine production

Five million thymocytes or splenocytes were resuspended in 3 ml of complete RPMI1640 medium [Sigma, supplemented with 10% FCS (Sigma), 100 U/ml penicillin–streptomycin (GE Healthcare), 2 mM l-glutamine (Sigma), 0.1 mM non-essential amino acid (Lonza), 1 mM sodium pyruvate (GE Healthcare), 55 μM β-mercaptoethanol (Sigma)] containing 50 ng/ml of phorbol 12-myristate 13-acetate (PMA; Sigma) and 500 ng/ml of ionomycin (Sigma) in the presence of Golgistop (BD Biosciences). Cells were harvested after 4.5 h incubation at 37 °C, and were stained with CD1d-tet as well as appropriate antibodies for surface markers. Cells were fixed with BD fixation buffer (BD Biosciences), and were stained for intracellular cytokines in 1 × BD perm buffer (BD Biosciences). Cytokine production was measured by LSRII or Fortessa (BD Biosciences).

### Generation of bone-marrow chimeric mice

Bone-marrow cells were incubated with biotinylated anti-CD3e (145-2C11; BD Biosciences) and anti-CD90.2 (53-2.1; BD Biosciences), followed by negative depletion with streptavidin beads (BD Biosciences). T-cell-depleted bone-marrow cells from *Mazr*^+/+^*Thpok*^+/GFP^*Lck*-Cre or *Mazr*^f/f^*Thpok*^+/GFP^*Lck*-Cre mice (both CD45.2^+^) were mixed at a 1:1 ratio with CD45.1^+^ bone-marrow cells, and a total of 4 × 10^6^ cells were injected into the tail vein of lethally irradiated (8.25 Gy) CD45.1^+^ congenic mice. Eight weeks after transplantation, reconstituted mice were sacrificed, and iNKT cell development was analyzed.

### α-GalCer treatment

α-GalCer (KRN-7000, Funakoshi) was dissolved in vehicle (5.6% sucrose, 0.75% l-histidine, and 0.5% Tween-20) to make 200 μg/ml stock solution. The stock solution was diluted 1:20 with PBS (Sigma), and mice were administrated a single intravenous injection of α-GalCer at 100 ng/g of body weight. Control mice were administrated an equivalent volume of vehicle diluted in PBS. Ninety minutes after administration mice were euthanized, and single-cell suspensions of splenocytes and hepatic lymphocytes were prepared, incubated in complete RPMI-1640 medium containing GolgiPlug (1:1000) and GolgiStop (1:1500) (BD Biosciences) for 2 h at 37 °C, and analyzed for cytokine production in iNKT cells. Twenty-four hours after α-GalCer administration sera were prepared, and the levels of alanine aminotransferase (ALT) and aspartate aminotransferase (AST) were measured enzymatically (Roche Diagnostics, Mannheim, Germany).

### Statistical analysis

An unpaired Student’s *t* test was performed for statistical analysis using Prism 6 software (GraphPad), unless otherwise indicated. The *p* values were defined as follows: *p < 0.05; **p < 0.01; ***p < 0.001; n.s., not significant.

## Results

### Loss of MAZR leads to an increase in CD44^+^NK1.1^−^ stage 2 iNKT cells

**Fig. 1:**
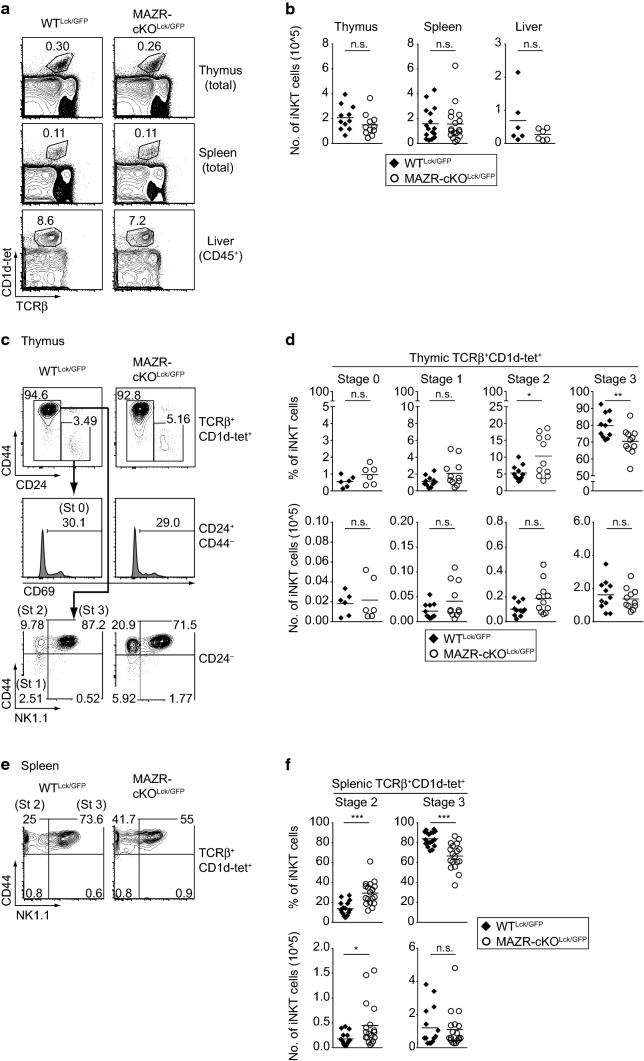
Deletion of MAZR in T cells leads to an increase in the number of stage 2 iNKT cells. **a** Flow cytometry analysis showing murine CD1d tetramers loaded with PBS-57 (CD1d-tet) staining and TCRβ expression on thymocytes, splenocytes, and CD45^+^ gated hepatic lymphocytes isolated from WT^Lck/GFP^ and MAZR-cKO^Lck/GFP^ mice. Numbers indicate the percentages of iNKT cells. **b** Diagrams showing the total cell numbers of iNKT cells in thymi, spleens, and livers of WT^Lck/GFP^ and MAZR-cKO^Lck/GFP^ mice. **c** Flow cytometry analysis showing CD44 and CD24 expression on TCRβ^+^CD1d-tet^+^ thymocytes (upper panel), CD69 expression on TCRβ^+^CD1d-tet^+^CD44^−^CD24^+^ thymocytes (middle panel) and CD44 and NK1.1 expression on TCRβ^+^CD1d-tet^+^CD24^−^ thymocytes (lower panel) isolated from WT^Lck/GFP^ and MAZR-cKO^Lck/GFP^ mice. Gating regions for TCRβ^+^CD1d-tet^+^CD44^−^CD24^+^ and TCRβ^+^CD1d-tet^+^CD24^−^ thymocytes are indicated in upper panel, while regions for stage 0 (St 0; CD24^+^CD44^−^CD69^+^), stage 1 (St 1; CD24^−^CD44^−^NK1.1^−^), stage 2 (St 2; CD24^−^CD44^+^NK1.1^−^), and stage 3 (St 3; CD24^−^CD44^+^NK1.1^+^) iNKT cells are shown in middle and lower panels. Numbers indicate the percentages of cells within the respective regions. **d** Diagrams showing the percentages (upper panel) and total cell numbers (lower panel) of thymic stage 0 (far left panel), stage 1 (left panel), stage 2 (right panel), and stage 3 (far right panel) iNKT cells in WT^Lck/GFP^ and MAZR-cKO^Lck/GFP^ mice. **e** Flow cytometry analysis showing CD44 and NK1.1 expression on TCRβ^+^CD1d-tet^+^ splenocytes isolated from WT^Lck/GFP^ and MAZR-cKO^Lck/GFP^ mice. Gating regions for stage 2 (St 2; CD44^+^NK1.1^−^) and stage 3 (St 3; CD44^+^NK1.1^+^) iNKT cells are shown in the plots. Numbers indicate the percentages of cells within the respective regions. **f** Diagrams showing the percentages (upper panel) and total cell numbers (lower panel) of splenic stage 2 (left panel) and stage 3 (right panel) iNKT cells in WT^Lck/GFP^ and MAZR-cKO^Lck/GFP^ mice. **a**, **c**, **e** Data are representative of at least ten mice analyzed in at least five independent experiments, except for thymic stage 0 iNKT cell analysis (**c**), where data are representative of six mice analyzed in five independent experiments. **b**,**d**, **f** Each dot represents one mouse. Horizontal bars indicate mean values

To test the role of MAZR during iNKT cell development, we analyzed mice with a T-cell-specific deletion of MAZR using the *Lck*-Cre deleter strain (*Mazr*^f/f^*Lck*-Cre) [35, 38]. In addition, since MAZR represses ThPOK expression in cytotoxic T cells [24, 25], and since ThPOK expression is dynamically modulated during iNKT cell development [28, 29], we introduced a *Thpok*-GFP knock-in reporter allele (*Thpok*^+/GFP^) into MAZR-deficient mice, allowing us to monitor *Thpok* expression by GFP expression [37]. In the end, *Mazr*^f/f^*Thpok*^+/GFP^*Lck*-Cre and control *Mazr*^+/+^*Thpok*^+/GFP^*Lck*-Cre mice (for simplicity designated MAZR-cKO^Lck/GFP^ and WT^Lck/GFP^ throughout the manuscript, respectively) were used to investigate the impact of MAZR deficiency on iNKT cell development. The total numbers of iNKT cells in thymus, spleen and liver were similar between WT^Lck/GFP^ and MAZR-cKO^Lck/GFP^ mice (Fig. 1a, b). However, the percentages of thymic stage 2 (CD24^−^CD44^+^NK1.1^–^) iNKT cells were significantly increased in MAZR-cKO^Lck/GFP^ mice compared to WT^Lck/GFP^ mice, along with a corresponding reduction in the percentage of stage 3 (CD24^−^CD44^+^NK1.1^+^) iNKT cells (Fig. 1c, d). A similar increase in the percentage of stage 2 iNKT cells was also observed in spleen and liver of MAZR-cKO^Lck/GFP^ mice (Fig. 1e, f; Fig. S1a, S1b). Moreover, the total number of splenic stage 2 iNKT cells was elevated in the absence of MAZR. These results suggest that MAZR negatively regulates the differentiation of stage 2 iNKT cells.

**Fig. 2:**
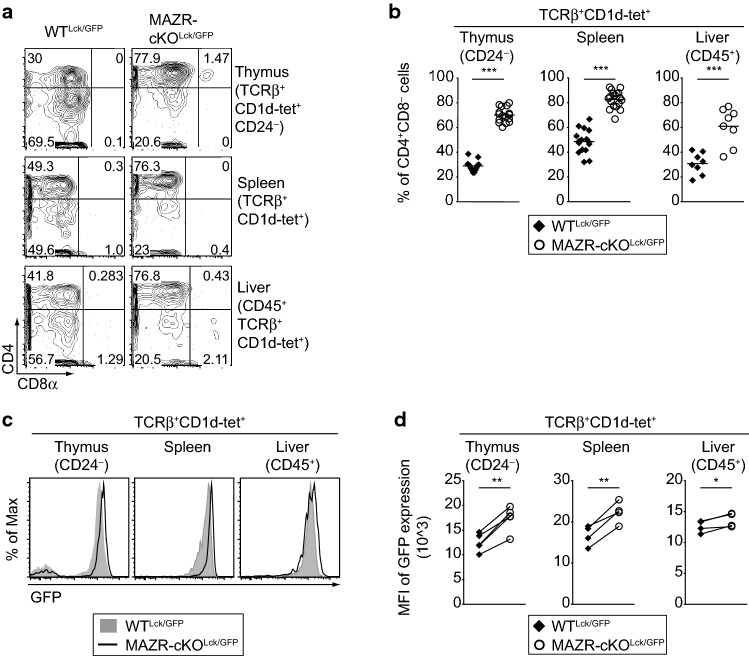
Deletion of MAZR in T cells leads to an increased proportion of CD4^+^CD8^−^ iNKT subset and elevated expression of ThPOK in iNKT cells. **a** Flow cytometry analysis showing CD4 and CD8α expression on TCRβ^+^CD1d-tet^+^CD24^−^ thymocytes (upper panel), TCRβ^+^CD1d-tet^+^ splenocytes (middle panel) and CD45^+^TCRβ^+^CD1d-tet^+^ hepatic lymphocytes (lower panel) isolated from WT^Lck/GFP^ and MAZR-cKO^Lck/GFP^ mice. Numbers indicate the percentages of cells within the respective regions. Data are representative of at least ten mice analyzed in at least five independent experiments (thymocytes and splenocytes), and of eight mice analyzed in 4 independent experiments (hepatic lymphocytes). **b** Diagrams showing the percentages of CD4^+^CD8α^−^ cells within TCRβ^+^CD1d-tet^+^CD24^−^ thymocytes (left panel), TCRβ^+^CD1d-tet^+^ splenocytes (middle panel) and CD45^+^TCRβ^+^CD1d-tet^+^ hepatic lymphocytes (right panel) isolated from WT^Lck/GFP^ and MAZR-cKO^Lck/GFP^ mice. Each dot represents one mouse. Horizontal bars indicate mean values. **c** Histograms showing GFP expression (i.e., *Thpok* expression) in TCRβ^+^CD1d-tet^+^CD24^−^ thymocytes (left panel), TCRβ^+^CD1d-tet^+^ splenocytes (middle) and CD45^+^TCRβ^+^CD1d-tet^+^ hepatic lymphocytes (right) isolated from WT^Lck/GFP^ and MAZR-cKO^Lck/GFP^ mice. Data are representative of at least ten mice analyzed in at least five independent experiments (thymocytes), and of eight mice analyzed in four independent experiments (splenocytes and hepatic lymphocytes). **d** Diagrams showing mean fluorescent intensity (MFI) of GFP expression in TCRβ^+^CD1d-tet^+^CD24^−^ thymocytes (left panel), TCRβ^+^CD1d-tet^+^ splenocytes (middle panel), and CD45^+^TCRβ^+^CD1d-tet^+^ hepatic lymphocytes (right panel) isolated from WT^Lck/GFP^ and MAZR-cKO^Lck/GFP^ mice. Each dot represents the average value of an individual experiment, in which 2–3 mice were analyzed per group. The lines indicate paired experiments. A paired two-tailed Student’s *t* test was performed for statistical analysis

Previous studies have demonstrated that the majority (approx. 80%) of stage 2 iNKT cells display a CD4^+^CD8^−^ phenotype and express ThPOK at the highest level among iNKT subsets [16, 29]. We, therefore, examined CD4, CD8, and GFP (i.e., *Thpok*) expression in MAZR-cKO^Lck/GFP^ iNKT cells. Notably, MAZR-cKO^Lck/GFP^ mice displayed an increased percentage of CD4^+^CD8^−^ iNKT cells in all analyzed tissues when compared with WT^Lck/GFP^ mice (Fig. 2a, b). Moreover, the level of GFP (i.e., of *Thpok*) expression was increased in the absence of MAZR (Fig. 2c, d). Thus, the deletion of MAZR leads to an increase in stage 2 iNKT cells, concurrent with the enhanced expression of CD4 and ThPOK in iNKT cells.

### Increased numbers of iNKT2 cells in the absence of MAZR

**Fig. 3:**
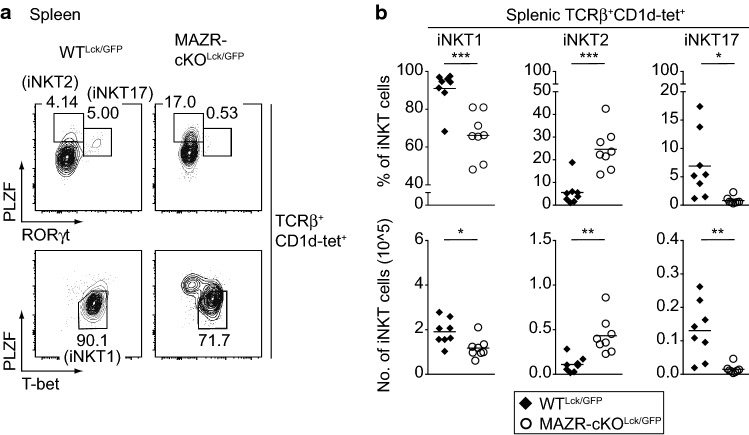
Distribution of iNKT cell subsets is altered in the absence of MAZR. **a** Flow cytometry analysis showing PLZF and RORγt expression (upper panel) and PLZF and T-bet expression (lower panel) in TCRβ^+^CD1d-tet^+^ splenocytes isolated from WT^Lck/GFP^ and MAZR-cKO^Lck/GFP^ mice. Numbers indicate the percentages of splenic T-bet^hi^PLZF^lo^ iNKT1, PLZF^hi^RORγt^−^ iNKT2, and PLZF^mid^RORγt^+^ iNKT17 cells. Data are representative of eight mice analyzed in four independent experiments. **b** Diagrams showing the percentages (upper panel) and numbers (lower panel) of splenic iNKT1 (left panel), iNKT2 (middle panel) and iNKT17 (right panel) cells in WT^Lck/GFP^ and MAZR-cKO^Lck/GFP^ mice. Each dot represents one mouse. Horizontal bars indicate mean values

According to the “lineage diversification” model, iNKT cells can be categorized into three distinct subsets based on the expression pattern of several transcription factors, including T-bet, PLZF and RORγt; iNKT1 (T-bet^hi^PLZF^lo^), iNKT2 (PLZF^hi^RORγt^−^) and iNKT17 (PLZF^mid^RORγt^+^) cells [15]. Since the deletion of MAZR led to an increase in stage 2 iNKT subsets, which includes iNKT2 and iNKT17 cells [15], we assessed the expression pattern of these signature transcription factors in MAZR-cKO^Lck/GFP^ iNKT cells. This revealed that the percentage and numbers of iNKT2 cells in the thymus and spleen were increased in the absence of MAZR, accompanied with a reduction in the number of splenic iNKT1 and iNKT17 cells in MAZR-cKO^Lck/GFP^ mice (Fig. 3a, b; Fig. S2a, S2b). In addition to the PLZF^hi^RORγt^−^ expression phenotype, iNKT2 cells are also characterized by the expression of several surface markers including CD4, CD27, and IL-17RB as well as the transcription factor GATA-binding protein 3 (GATA3) [15, 16]. MAZR-cKO^Lck/GFP^ iNKT2 cells expressed all of those markers at similar levels in comparison with WT^LCK/GFP^ iNKT2 cells (Fig. S3a, S3b). These data suggest that the increase in stage 2 iNKT subsets in the absence of MAZR was due to enhanced iNKT2 cell differentiation. Furthermore, these results indicate that MAZR negatively regulates iNKT2 cell differentiation and that MAZR is required for splenic iNKT1 and iNKT17 cell differentiation.

### iNKT cell-intrinsic alterations in the absence of MAZR

**Fig. 4:**
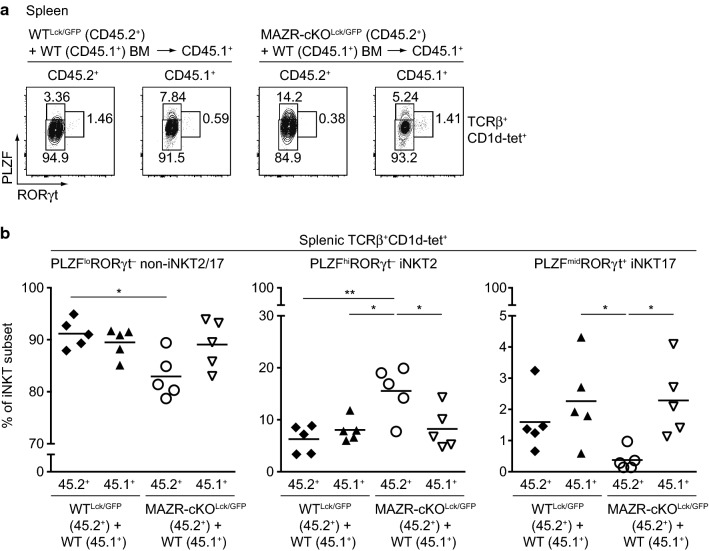
Altered iNKT cell development by loss of MAZR is due to iNKT cell-intrinsic defects. **a** Flow cytometry analysis showing PLZF and RORγt expression in CD45.2^+^ and CD45.1^+^ subsets of TCRβ^+^CD1d-tet^+^ splenocytes isolated from BM chimeric CD45.1^+^ mice. CD45.1^+^ recipient mice received either WT^Lck/GFP^ (CD45.2^+^) or MAZR-cKO^Lck/GFP^ (CD45.2^+^) BM cells that had been mixed at a 1:1 ratio with wild-type (WT, CD45.1^+^) BM cells. Data are representative of five mice analyzed in two independent experiments. **b** Diagram showing the percentage of splenic PLZF^lo^RORγt^−^ non-iNKT2/17 (left panel), PLZF^hi^RORγt^−^ iNKT2 (middle panel), and PLZF^mid^RORγt^+^ iNKT17 (right panel) cells within CD45.2^+^ (45.2^+^) and CD45.1^+^ (45.1^+^) subsets of TCRβ^+^CD1d-tet^+^ splenocytes isolated from BM chimeric CD45.1^+^ mice generated as described in **a**. Each dot represents one mouse. Horizontal bars indicate mean values. A one-way ANOVA followed by Tukey multiple comparison test was used for statistical analysis. Differences that did not reach a statistically significant level (i.e., *p* ≥ 0.05) are not indicated

The *Lck*-Cre strain initiates deletion at the DN2 to DN3 transition of thymocyte development [38], and MAZR is thereby deleted in all T-cell subsets beyond this developmental stage. It is, therefore, possible that the increase in iNKT2 cells in MAZR-cKO^Lck/GFP^ mice is due to alterations in non-iNKT T-cell subsets that lead to changes in iNKT cells. To determine whether loss of MAZR led to iNKT cell-intrinsic changes, competitive bone-marrow (BM) reconstitution experiments were performed. For this, WT^Lck/GFP^ (CD45.2^+^) or MAZR-cKO^Lck/GFP^ (CD45.2^+^) BM cells were mixed with congenic (CD45.1^+^) WT BM cells at a 1:1 ratio and injected into lethally irradiated CD45.1^+^ mice. 8–10 weeks later, thymic and splenic iNKT cell subsets in reconstituted BM chimeric mice were analyzed (Fig. S4a). Similar to the phenotype observed in MAZR-cKO^Lck/GFP^ mice, splenic iNKT cells in the MAZR-cKO^Lck/GFP^ compartment of BM chimeric mice displayed an increased fraction of iNKT2 cells, with a corresponding reduction in the percentage of the other subsets [although the difference in iNKT17 cell percentage between WT^Lck/GFP^ and MAZR-cKO^Lck/GFP^ compartments reached statistical significance (*p* = 0.0298) only with an unpaired two-tailed Student’s *t* test] (Fig. 4a, b). In addition, there was also a tendency that thymic MAZR-deficient iNKT2 subsets were enhanced in BM chimeric mice (Fig. S4b, S4c). Together, these results suggest that the alterations in the distribution of iNKT subsets in MAZR-cKO^Lck/GFP^ are due to iNKT cell-intrinsic defects.

### Deletion of MAZR leads to alterations in iNKT cell cytokine expression

**Fig. 5:**
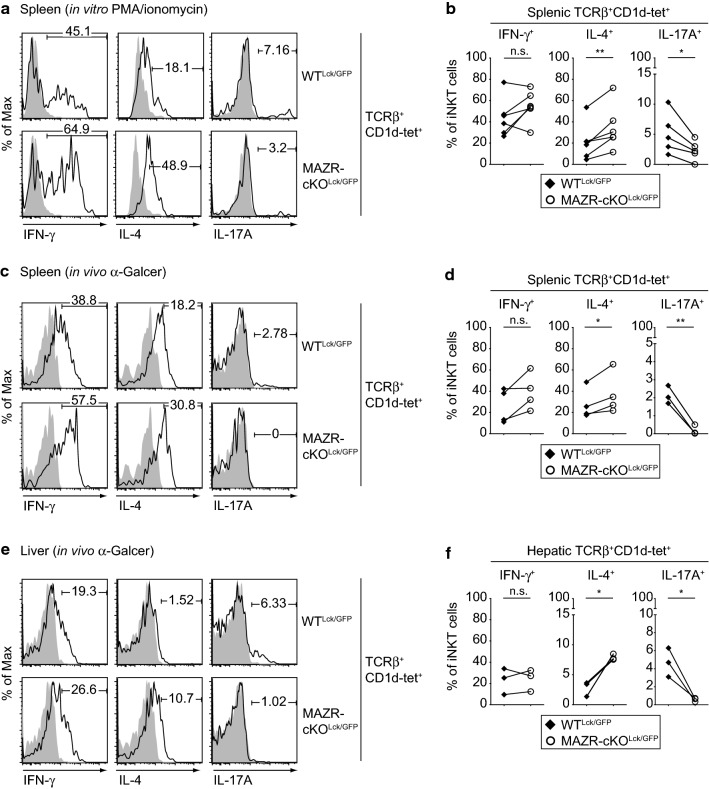
MAZR-deficient iNKT cells display altered cytokine production. **a** Histograms showing IFN-γ (left panel), IL-4 (middle panel), and IL-17A (right panel) expression in TCRβ^+^CD1d-tet^+^ splenocytes isolated from WT^Lck/GFP^ (upper panel) and MAZR-cKO^Lck/GFP^ (lower panel) mice. Splenocytes were stimulated with PMA and ionomycin for 4.5 h in the presence of GolgiStop. Filled histograms show the expression level of each cytokine in unstimulated WT^Lck/GFP^ TCRβ^+^CD1d-tet^+^ splenocytes. Numbers indicate the percentages of cells within the respective regions. Data are representative of more than ten mice analyzed in at least five independent experiments. **b** Diagrams showing the percentage of IFN-γ^+^ (left panel), IL-4^+^ (middle panel), and IL-17A^+^ (right panel) PMA/ionomycin-stimulated splenic iNKT cells isolated from WT^Lck/GFP^ and MAZR-cKO^Lck/GFP^ mice. **c** Histograms showing IFN-γ (left panel), IL-4 (middle panel), and IL-17A (right panel) expression in TCRβ^+^CD1d-tet^+^ splenocytes isolated from α-GalCer-treated WT^Lck/GFP^ (upper panel) and MAZR-cKO^Lck/GFP^ (lower panel) mice. Filled histograms show the expression level of each cytokine in splenic iNKT cells from vehicle-treated WT^Lck/GFP^ mice. **d** Diagrams showing the percentage of IFN-γ^+^ (left panel), IL-4^+^ (middle panel), and IL-17A^+^ (right panel) splenic iNKT cells isolated from α-GalCer-treated WT^Lck/GFP^ and MAZR-cKO^Lck/GFP^ mice. **e** Histograms showing IFN-γ (left panel), IL-4 (middle panel), and IL-17A (right panel) expression in TCRβ^+^CD1d-tet^+^ hepatic lymphocytes isolated from α-GalCer-treated WT^Lck/GFP^ (upper panel) and MAZR-cKO^Lck/GFP^ (lower panel) mice. Filled histograms show the expression level of each cytokine in hepatic iNKT cells from vehicle-treated WT^Lck/GFP^ mice. **f** Diagrams showing the percentage of IFN-γ^+^ (left panel), IL-4^+^ (middle panel), and IL-17A^+^ (right panel) hepatic iNKT cells isolated from α-GalCer-treated WT^Lck/GFP^ and MAZR-cKO^Lck/GFP^ mice. **c**, **e** Splenocytes and hepatic lymphocytes were isolated 1.5 h after α-GalCer administration, and were stained for intracellular cytokines after incubation with GolgiStop and GolgiPlug. Numbers indicate the percentages within the respective quadrants. Data are representative of 6–7 mice analyzed in 3–4 independent experiments. **b**, **d**, **f** Each dot represents the average value of an individual experiment, in which 1–3 mice were analyzed per group. The lines indicate paired experiments. A paired two-tailed Student’s *t* test was performed for statistical analysis

iNKT cells rapidly produce various cytokines upon stimulation [4, 20]. iNKT2 and iNKT17 cells mainly secrete IL-4 and IL-17A, respectively, whereas iNKT1 cells produce both IFN-γ and IL-4 [15]. To determine cytokine production in the absence of MAZR, we activated thymic and splenic iNKT cells isolated from WT^Lck/GFP^ and MAZR-cKO^Lck/GFP^ mice with PMA/ionomycin and measured IFN-γ, IL-4, and IL-17A expression by intracellular staining. In agreement with the increase in iNKT2 subsets in the absence of MAZR, the MAZR-cKO^Lck/GFP^ iNKT cell population contained a higher percentage of IL-4-producing cells compared to the WT^Lck/GFP^ iNKT cell population (Fig. 5a, b; Fig. S5a, S5b). In addition, splenic iNKT cells produced less IL-17A (Fig. 5a, b), which is also consistent with the observed reduction of splenic iNKT17 cell numbers in the absence of MAZR (Fig. 3). Of note, the analysis of cytokine production in NK1.1^+^ iNKT cells revealed that the proportion of IFN-γ^+^ cells was increased in the absence of MAZR (Fig. S5c, S5d). This suggests that MAZR-cKO^Lck/GFP^ iNKT1 cells express a higher amount of IFN-γ despite the reduction in their number. Next, we assessed cytokine production of MAZR-deficient iNKT cells upon in vivo stimulation. Therefore, α-GalCer was injected intravenously into WT^Lck/GFP^ and MAZR-cKO^Lck/GFP^ mice and cytokine production of splenic and hepatic iNKT cells was determined 90 min post-injection. In line with our findings in in vitro PMA/ionomycin-stimulated iNKT cells (Fig. 5a, b), splenic and hepatic MAZR-cKO^Lck/GFP^ iNKT cells from mice receiving α-GalCer produced more IL-4, while IL-17A production was reduced in comparison with WT^Lck/GFP^ iNKT cells (Fig. 5c–f). Moreover, there was a tendency (*p* = 0.0911) that the proportion of IFN-γ^+^ cells in splenic MAZR-cKO^Lck/GFP^ iNKT cells was increased. This is consistent with an increased IFN-γ production in NK1.1^+^ MAZR-cKO^Lck/GFP^ iNKT cells in vitro (Fig. S5c, S5d), since intravenous α-GalCer injection was shown to preferentially activate iNKT1 cells in the spleen [39]. Together, these data suggest that MAZR is required for the proper regulation of cytokine production in iNKT cells both upon in vitro and in vivo stimulation.

### Increased Egr2 expression during iNKT cell development by loss of MAZR

**Fig. 6:**
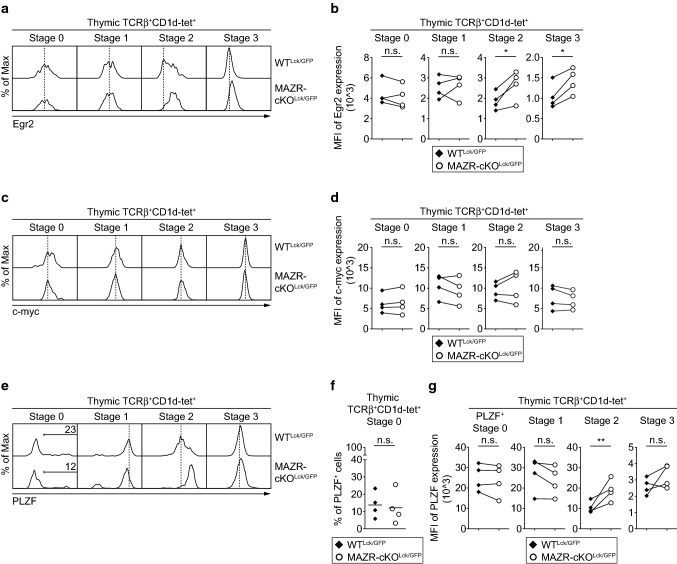
Enhanced Egr2 expression in thymic MAZR-deficient iNKT cells. **a** Histograms showing Egr2 expression in thymic stage 0 (CD24^+^CD44^−^CD69^+^), stage 1 (CD24^−^CD44^−^NK1.1^−^), stage 2 (CD24^−^CD44^+^NK1.1^−^) and stage 3 (CD24^−^CD44^+^NK1.1^+^) iNKT cells isolated from WT^Lck/GFP^ and MAZR-cKO^Lck/GFP^ mice. Dotted lines indicate the peaks of Egr2 expression in WT^Lck/GFP^ iNKT cells at the individual stages. **b** Diagrams showing mean fluorescent intensity (MFI) of Egr2 expression in each developmental subset of thymic iNKT cells isolated from WT^Lck/GFP^ and MAZR-cKO^Lck/GFP^ mice. **c** Histograms showing c-myc expression in each developmental subset of thymic iNKT cells (identified as described in **a**) isolated from WT^Lck/GFP^ and MAZR-cKO^Lck/GFP^ mice. Dotted lines indicate the peaks of c-myc expression in WT^Lck/GFP^ iNKT cells at the individual stages. **d** Diagrams showing MFI of c-myc expression in each developmental subset of thymic iNKT cells isolated from WT^Lck/GFP^ and MAZR-cKO^Lck/GFP^ mice. **e** Histograms showing PLZF expression in each developmental subset of thymic iNKT cells (identified as described in **a**) isolated from WT^Lck/GFP^ and MAZR-cKO^Lck/GFP^ mice. Regions indicate PLZF^+^ cells within stage 0 iNKT cells, and the numbers above show the proportion of the cells. Dotted lines indicate the peaks of PLZF expression in stage 1–3 WT^Lck/GFP^ iNKT cells. **f** Diagram showing the percentage of PLZF^+^ cells within thymic stage 0 iNKT cells isolated from WT^Lck/GFP^ and MAZR-cKO^Lck/GFP^ mice. Each dot represents one mouse. Horizontal bars indicate mean values. **g** Diagrams showing MFI of PLZF expression in PLZF^+^stage 0 as well as stage 1–3 thymic iNKT cells isolated from WT^Lck/GFP^ and MAZR-cKO^Lck/GFP^ mice. **a**, **c**, **e** Data are representative of four mice analyzed in four independent experiments. **b**, **d**, **g** Each dot represents one mouse. The lines indicate paired experiments. A paired two-tailed Student’s *t* test was performed for statistical analysis

During positive selection, a strong TCR signal induces high-level expression of Egr2 in iNKT cell precursors [7]. Subsequently, Egr2 initiates the acquisition of effector properties via directly inducing PLZF expression, whereas the proliferative expansion of iNKT cell precursors is mediated by c-myc [7–11]. Previous studies have demonstrated that subtle changes in Egr2 and PLZF expression levels during the course of iNKT cell development have profound impact on iNKT cell subset differentiation [34, 40]. To examine whether MAZR regulates iNKT cell subset differentiation through the regulation of Egr2, c-myc and PLZF expression, we analyzed the expression of these molecules at each developmental stage of MAZR-cKO^Lck/GFP^ iNKT cells. The deletion of MAZR led to an increase in Egr2 expression at stage 2 and 3 iNKT cells in comparison with WT cells, whereas there was no alteration in c-myc expression in MAZR-cKO^Lck/GFP^ cells (Fig. 6a–d). In addition, stage 2 MAZR-cKO^Lck/GFP^ iNKT cells displayed enhanced PLZF expression compared to the corresponding WT^Lck/GFP^ iNKT cell subset, most likely due to the enlargement of the iNKT2 cell subset (Fig. 6e–g). However, despite the increase in Egr2 expression in stage 3 MAZR-cKO^Lck/GFP^ iNKT cells, there was no alteration in the expression level of PLZF in the cells (Fig. 6e, f). Thus, these data suggest that MAZR negatively regulates Egr2 expression at stage 2 and stage 3 of iNKT cell development.

## Discussion

In this study, we investigated the role of the transcription factor MAZR in the development and effector function of iNKT cells. We observed an increase in the number of thymic and splenic iNKT2 cells in MAZR-cKO^Lck/GFP^ mice, while splenic iNKT1 and iNKT17 cells were reduced. Moreover, BM reconstitution experiments indicated that the alteration in iNKT cell development in the absence of MAZR occurred in an iNKT cell-intrinsic manner. The increase in iNKT2 cells was also accompanied with elevated CD4, ThPOK, and Egr2 expression in MAZR-cKO^Lck/GFP^ iNKT cells, indicating that MAZR regulates ThPOK and CD4 expression not only in conventional T cells [24, 25], but also in iNKT cells. Thus, our data demonstrate an important role of MAZR for the regulation of iNKT cell development and subset differentiation. It is unlikely that the alterations in the distribution of iNKT cell subsets observed in MAZR-cKO^Lck/GFP^ mice were due to changes in the survival and/or proliferation of particular iNKT cell subsets, since there was no difference in the proportion of Annexin V^+^ cells or BrdU incorporating cells at the various classical CD44/NK1.1 developmental stages of iNKT cells (Fig. S6). A normal incorporation of BrdU is also in line with our data showing that there is no change in the expression of c-myc, known to be important for iNKT cell proliferation [10, 11], in MAZR-deficient iNKT cells. We, therefore, rather conclude that MAZR controls iNKT cell subset differentiation via regulating iNKT cell subset fate decisions and/or the subsequent differentiation processes of iNKT cell subsets.

Consistent with the alteration in iNKT cell subset differentiation, MAZR-deficient iNKT cells displayed enhanced production of IL-4, along with a reduction in IL-17A production. This was also observed upon injection of α-GalCer, which is a well-established model of acute hepatitis [41, 42]. Since IL-4 and IL-17A produced by iNKT cells have detrimental as well as protective functions in this model, respectively [43, 44], we also studied whether the observed changes in cytokine production lead to hepatic damage in WT^Lck/GFP^ and MAZR-cKO^Lck/GFP^ mice. However, serum levels of alanine aminotransferase (ALT) and aspartate aminotransferase (AST) in MAZR-cKO^Lck/GFP^ mice were comparable to those in WT^Lck/GFP^ mice (Fig. S7), suggesting that MAZR deficiency in T cells had no impact on α-GalCer-mediated acute hepatitis. Activated iNKT cells induce liver injury not only by secreting a broad spectrum of cytokines, but also via cytolytic proteins and through killer activation receptors [45, 46]. Thus, changes in some of these other mechanisms might compensate for alterations in cytokine expression upon loss of MAZR, thereby leading to a similar extent of liver injury as observed in WT^Lck/GFP^ mice.

In this study, we showed that MAZR negatively regulates Egr2 expression in stage 2 and 3 iNKT cells. Egr2 is induced upon the engagement of iNKT TCRs with glycolipid-loaded CD1d molecules, and initiates the acquisition of effector properties by directly inducing PLZF expression [7–9]. Moreover, recent studies showed that the modulation of TCR-signaling strength leads to alterations in iNKT cell subset differentiation, accompanied with changes in Egr2 expression levels in stage 1–3 iNKT cells [34, 47]. Since iNKT2 cells express the highest level of Egr2 followed by iNKT17 and iNKT1 cells [34], the increase in Egr2 expression in stage 2 MAZR-cKO^Lck/GFP^ iNKT cells might be due the enlargement of iNKT2 cell subset in the absence of MAZR. However, Egr2 expression was also increased in stage 3 MAZR-cKO^Lck/GFP^ iNKT cells, suggesting that MAZR is rather required to restrict Egr2 expression below a certain threshold level in stage 2 and stage 3 iNKT cells. Given the different expression levels of Egr2 among the three iNKT cell subsets, it might be conceivable that a fine-tuning of Egr2 expression levels by MAZR plays an important role for appropriate iNKT cell subset differentiation. Of note, MAZR protein is expressed at the highest level in stage 0 iNKT cells, and its expression levels are gradually downmodulated at the subsequent developmental stages (Fig. S8). Therefore, despite its rather low expression level, MAZR negatively regulates Egr2 expression at stage 2 and stage 3 iNKT cells. Further studies are required to address the mechanisms by which MAZR regulates Egr2 expression in iNKT cells and the impact of MAZR on developmental processes downstream of Egr2.

We previously demonstrated that MAZR is essential for the establishment and maintenance of ThPOK repression in MHC class I-signaled DP thymocytes and mature CD8^+^ T cells [24, 25]. Moreover, MAZR interacts with Runx proteins (i.e., Runx1 and Runx3) and cooperatively repress ThPOK during T-cell development [25]. In this study, we showed that MAZR controls ThPOK expression also in iNKT cells. Besides its role as key transcription factor for CD4^+^ T-cell commitment and lineage stability [48–51], ThPOK is also essential for proper iNKT cell subset differentiation [28–31]. In ThPOK-deficient mice, iNKT17 cells are increased, while iNKT2 cells are reduced [28, 29, 31]. Moreover, whereas WT iNKT cells do not express CD8, the deletion of ThPOK leads to the appearance of atypical CD4^−^CD8^+^ iNKT cells, concurrent with a complete loss of CD4^+^CD8^−^ subsets [28, 30, 31]. In contrast to the phenotype upon loss of ThPOK, we observed that iNKT2 cell differentiation was promoted in the absence of MAZR, which was accompanied with the inhibition of iNKT1 and iNKT17 cell differentiation. Furthermore, there was also an increase in the proportion of CD4^+^CD8^−^ iNKT cell subsets. Together, these data suggest antagonistic functions for ThPOK and MAZR during iNKT cell development. iNKT2 and iNKT17 cells express ThPOK at the highest and lowest levels among the three subsets, respectively [29]. Given that enforced ThPOK expression in iNKT cells led to the repression of RORγt, fine-tuning of ThPOK expression might be essential for appropriate iNKT cell subset differentiation, particularly for iNKT17 cell development [29, 31]. Thus, it is conceivable that the increase in ThPOK expression in MAZR-deficient iNKT cells contributes to the alteration in iNKT cell subset distribution. Since MAZR acts in synergy with Runx3 to repress ThPOK expression during CD8^+^ T-cell development [25], we also examined whether there is synergistic activity in the regulation of iNKT cell development by analyzing MAZR/Runx3 double-mutant mice (on a *Cd4*-Cre background) [25]. The combined deletion of MAZR and Runx3 led to a reduction of thymic iNKT cell numbers, and a similar tendency was also observed in Runx3 single-mutant mice (Fig. S9a, S9b). Notably, MAZR/Runx3 double-mutant iNKT cells expressed ThPOK at a higher level than WT control cells, and also showed a tendency of increased ThPOK expression compared to MAZR and Runx3 single-mutant cells (*p* = 0.0371 and 0.0494, respectively, with an unpaired two-tailed Student’s *t* test) (Fig S9c, S9d). Moreover, the combined loss of MAZR and Runx3 led to an increase in the percentage of iNKT2 cells, concurrent with a reduced percentage of iNKT17 cells (Fig. S9e, S9f). Together, these data indicate that MAZR and Runx3 synergistically repress ThPOK expression during iNKT cell development, thereby regulating iNKT cell subset differentiation. Therefore, we hypothesize that MAZR controls iNKT cell development through the regulation of Egr2 as well as ThPOK expression. However, it remains to be determined whether these two regulatory processes are connected with each other during iNKT cell development or whether these are two independent regulatory pathways. Further studies are required to elucidate how MAZR is integrated in the transcriptional program controlling iNKT cell subset differentiation, including whether MAZR-mediated fine-tuning of ThPOK expression is part of the processes determining iNKT cell fate.

Finally, we noticed that the alteration in iNKT cell subset differentiation in the absence of MAZR was more evident in the spleen than in the thymus, both under homeostatic conditions in MAZR-cKO^Lck/GFP^ mice as well as in competitive bone-marrow (BM) reconstitution experiments. Although the mechanism of peripheral iNKT cell development still remains largely unknown, a recent study has demonstrated that thymic iNKT cell subsets (particularly iNKT1 and iNKT17 cells) are largely tissue-resident and that peripheral iNKT cell subsets derive from a CCR7^+^ precursor subset of iNKT cells that emigrate from the thymus [52]. It is, therefore, possible that MAZR has a greater impact on iNKT cell subset differentiation in peripheral lymphoid organs compared to the thymus. In addition, the deletion of MAZR might lead to an alteration in the tissue residency of thymic iNKT cell subsets and/or in the homing capacity of mature iNKT cells. Of note, the phenotypic alterations of thymic MAZR-deficient iNKT cells in BM chimeric mice were even milder than the one observed in MAZR-cKO^Lck/GFP^ mice. This might be due to a differential impact of MAZR on thymic iNKT cell subset differentiation and/or the tissue residency of thymic iNKT cell subsets under a competitive environment in BM chimeric mice compared to homeostatic condition in MAZR-cKO^Lck/GFP^ mice. Further studies are required to address the role of MAZR for thymic iNKT cell development in a competitive BM chimeric setting.

Taken together, our study defines MAZR as an essential transcription factor regulating iNKT cell subset differentiation and effector function.

## Electronic supplementary material

Below is the link to the electronic supplementary material.

Supplementary material 1 (PDF 3465 kb)

## Abbreviations

ALT: Alanine aminotransferase
AST: Aspartate aminotransferase
BM: Bone marrow
CD1d-tet: Murine CD1d tetramer loaded with PBS-57
DN: Double-negative
DP: Double-positive
GATA3: GATA-binding protein 3
iNKT cells: Invariant natural killer T cells
MAZR: Myc-associated zinc-finger-related factor
PLZF: Promyelocytic leukemia zinc finger
RORγt: RAR-related orphan receptor gamma
Runx: Runt-related transcription factor
T-bet: T-box 21
TCR: T-cell receptor
ThPOK: T-helper-inducing POZ/Krueppel-like factor
α-GalCer: Alpha-galactosylceramide
