# Supplementary material for: The zinc-finger transcription factor MAZR regulates iNKT cell subset differentiation
Source: Cell Mol Life Sci. 2019 May 7;76(21):4391–404. doi: 10.1007/s00018-019-03119-z (PMC6803753; doi:10.1007/s00018-019-03119-z)
Supplement: Supplementary file 1 — Supplementary material 1 (PDF 3465 kb) [file 18_2019_3119_MOESM1_ESM.pdf]

Supplementary information for the manuscript

Full title: The zinc-finger transcription factor MAZR regulates iNKT cell subset differentiation

Authors: Maria Jonah Orola[1,5,7], Caroline Tizian[1,6,7], Ci Zhu[2], Liisa Andersen[1], Alexandra Franziska Gülich[1], Marlis Alteneder[1], Tatjana Stojakovic[3], Ursula Wiedermann[4], Michael Trauner[2], Wilfried Ellmeier[1], Shinya Sakaguchi[1]

Address correspondence to Dr. Shinya Sakaguchi at the Division of Immunobiology, Institute of Immunology, Medical University of Vienna, Lazarettgasse 19, 1090 Vienna, Austria. Phone: +43-1-40160-33297; Fax: +43-1-40160-933293

E-mail: shinya.sakaguchi@meduniwien.ac.at

Content:

Figure S1: The subset distribution of hepatic iNKT cells is altered in the absence of MAZR.

Figure S2: Deletion of MAZR leads to an alteration in thymic iNKT cell subset distribution.

Figure S3: MAZR-deficient iNKT2 cells display iNKT2 characteristics.

Figure S4: Thymic iNKT cell differentiation upon competitive bone marrow-reconstitution.

Figure S5: Cytokine production in thymic as well as NK1.1^{+} and NK1.1^{-} splenic iNKT cells.

Figure S6: Survival as well as proliferation of iNKT cells is intact in the absence of MAZR.

Figure S7: Loss of MAZR led to no alteration in the degree of liver injury upon α-GalCer challenge.

Figure S8: Expression pattern of MAZR during thymic iNKT cell development.

Figure S9: MAZR and Runx3 synergistically control thymic iNKT cell development.

Table S1: Antibodies used in this study.

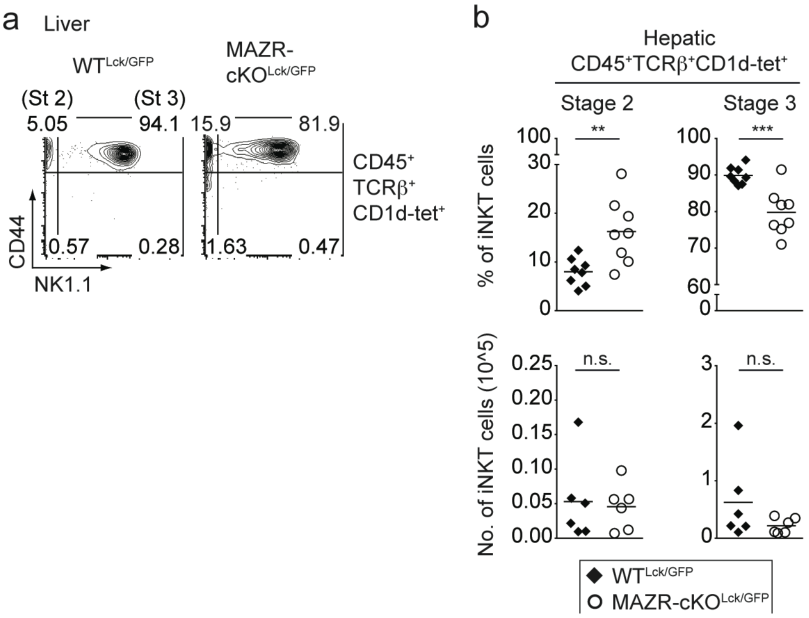

Fig. S1 The subset distribution of hepatic iNKT cells is altered in the absence of MAZR. **a** Flow cytometry analysis showing CD44 and NK1.1 expression on CD45$^+$TCRβ$^+$CD1d-tet$^+$ hepatic lymphocytes isolated from WT$^{Lck/GFP}$ and MAZR-cKO$^{Lck/GFP}$ mice. Gating regions for stage 2 (St 2; CD44$^+$NK1.1$^-$) and stage 3 (St 3; CD44$^+$NK1.1$^+$) iNKT cells are shown in the plots. Numbers indicate the percentages of cells within the respective regions. Data are representative of 8 mice analyzed in 4 independent experiments. **b** Diagrams showing the percentage (upper panel) and total cell numbers (lower panel) of hepatic stage 2 (left panel) and stage 3 (right panel) iNKT cells in WT$^{Lck/GFP}$ and MAZR-cKO$^{Lck/GFP}$ mice. Each dot represents one mouse. Horizontal bars indicate mean values.

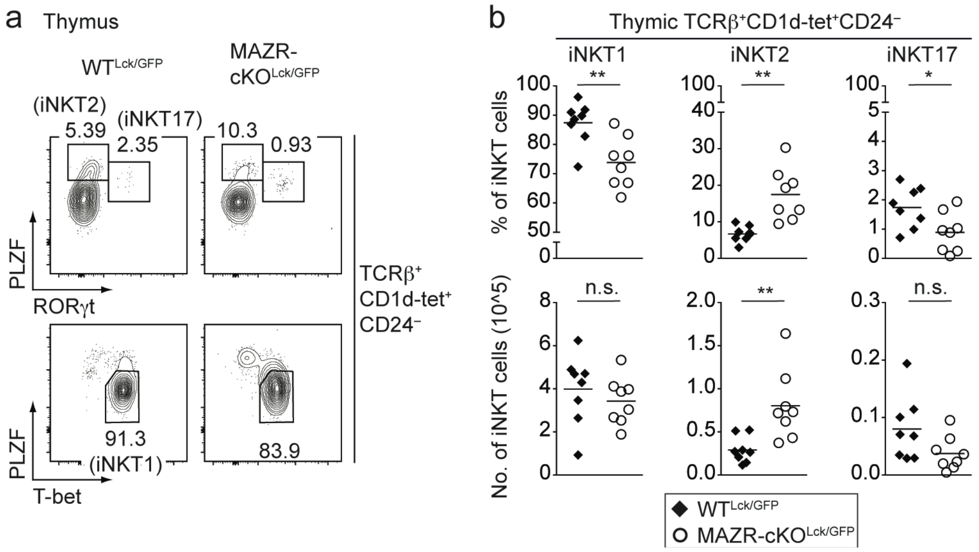

Fig. S2 Deletion of MAZR leads to an alteration in thymic iNKT cell subset distribution. **a** Flow cytometry analysis showing PLZF and Rorγt expression (upper panel) and PLZF and T-bet expression (lower panel) in $TCR\beta^{+}CD1d\text{-}tet^{+}CD24^{-}$ thymocytes isolated from $WT^{Lck/GFP}$ and MAZR-$cKO^{Lck/GFP}$ mice. Numbers indicate the percentages of thymic T-$bet^{hi}PLZF^{lo}$ iNKT1, $PLZF^{hi}ROR\gamma t^{-}$ iNKT2 and $PLZF^{mid}ROR\gamma t^{+}$ iNKT17 cells. Data are representative of 8 mice analyzed in 4 independent experiments. **b** Diagrams showing the percentages (upper panel) and numbers (lower panel) of thymic iNKT1 (left panel), iNKT2 (middle panel) and iNKT17 (right panel) cells in $WT^{Lck/GFP}$ and MAZR-$cKO^{Lck/GFP}$ mice. Each dot represents one mouse. Horizontal bars indicate mean values.

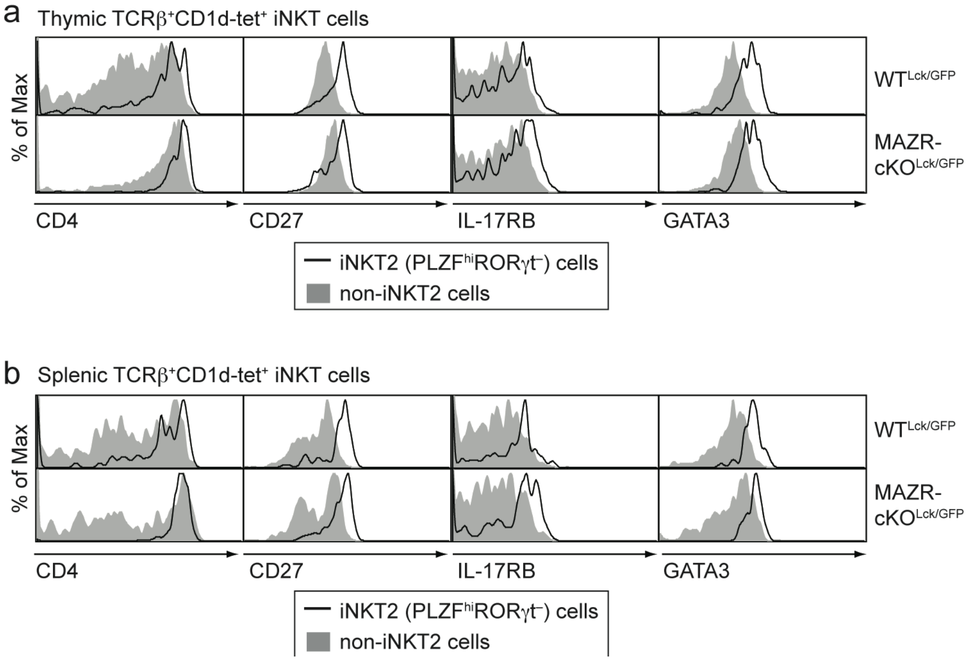

Fig. S3 MAZR-deficient iNKT2 cells display iNKT2 characteristics. **a** Histograms showing CD4, CD27, IL-17RB and GATA3 expression in thymic iNKT2 (PLZF^{hi}RORγt^{-}: open histograms) and non-iNKT cells (filled histograms) from WT$^{Lck/GFP}$ and MAZR-cKO$^{Lck/GFP}$ mice. **b** Histograms showing CD4, CD27, IL-17RB and GATA3 expression in splenic iNKT2 (PLZF^{hi}RORγt^{-}: open histograms) and non-iNKT cells (filled histograms) from WT$^{Lck/GFP}$ and MAZR-cKO$^{Lck/GFP}$ mice. **a,b** Data are representative of 4 mice analyzed in 2 independent experiments.

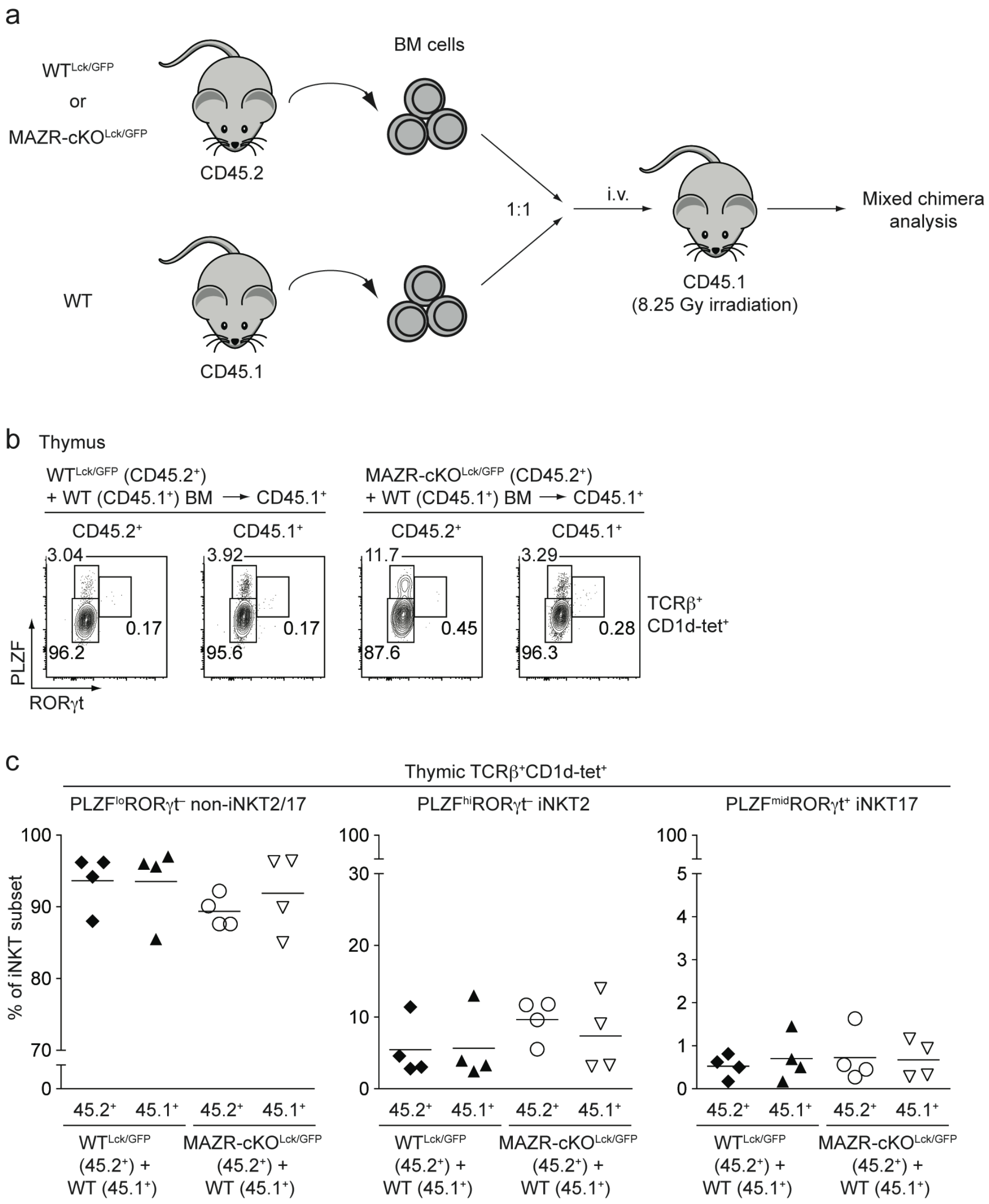

Fig. S4 Thymic iNKT cell differentiation upon competitive bone marrow-reconstitution. **a** Schematic figure showing the procedure of competitive bone marrow-reconstitution experiments. Bone marrow (BM) cells from WT$^{Lck/GFP}$ (CD45.2$^+$) or MAZR-cKO$^{Lck/GFP}$ (CD45.2$^+$) mice were mixed 1:1 with CD45.1$^+$ congenic bone marrow cells, and were intravenously injected into lethally irradiated CD45.1$^+$ mice. Eight to ten weeks later iNKT cell development of the mixed chimeric mice were analyzed. **b** Flow cytometry analysis showing PLZF and RORγt expression in CD45.2$^+$ and CD45.1$^+$ subsets of TCRβ$^+$CD1d-tet$^+$ thymocytes isolated from BM chimeric CD45.1$^+$ mice generated as describe above. Data are representative of 4 mice analyzed in 2 independent experiments. **c** Diagram showing the percentage of thymic PLZF^{lo}RORγt$^-$ non-iNKT2/17 (left panel), PLZF^{hi}RORγt$^-$ iNKT2 (middle panel) and PLZF^{mid}RORγt$^+$ iNKT17 (right panel) cells within CD45.2$^+$ (45.2$^+$) and CD45.1$^+$ (45.1$^+$) subsets of TCRβ$^+$CD1d-tet$^+$ thymocytes isolated from BM chimeric CD45.1$^+$ mice generated as described above. Each dot represents one mouse. Horizontal bars indicate mean values. A one-way ANOVA followed by Tukey's multiple comparison test was used for statistical analysis. No comparison between two groups reached a statistically significant level.

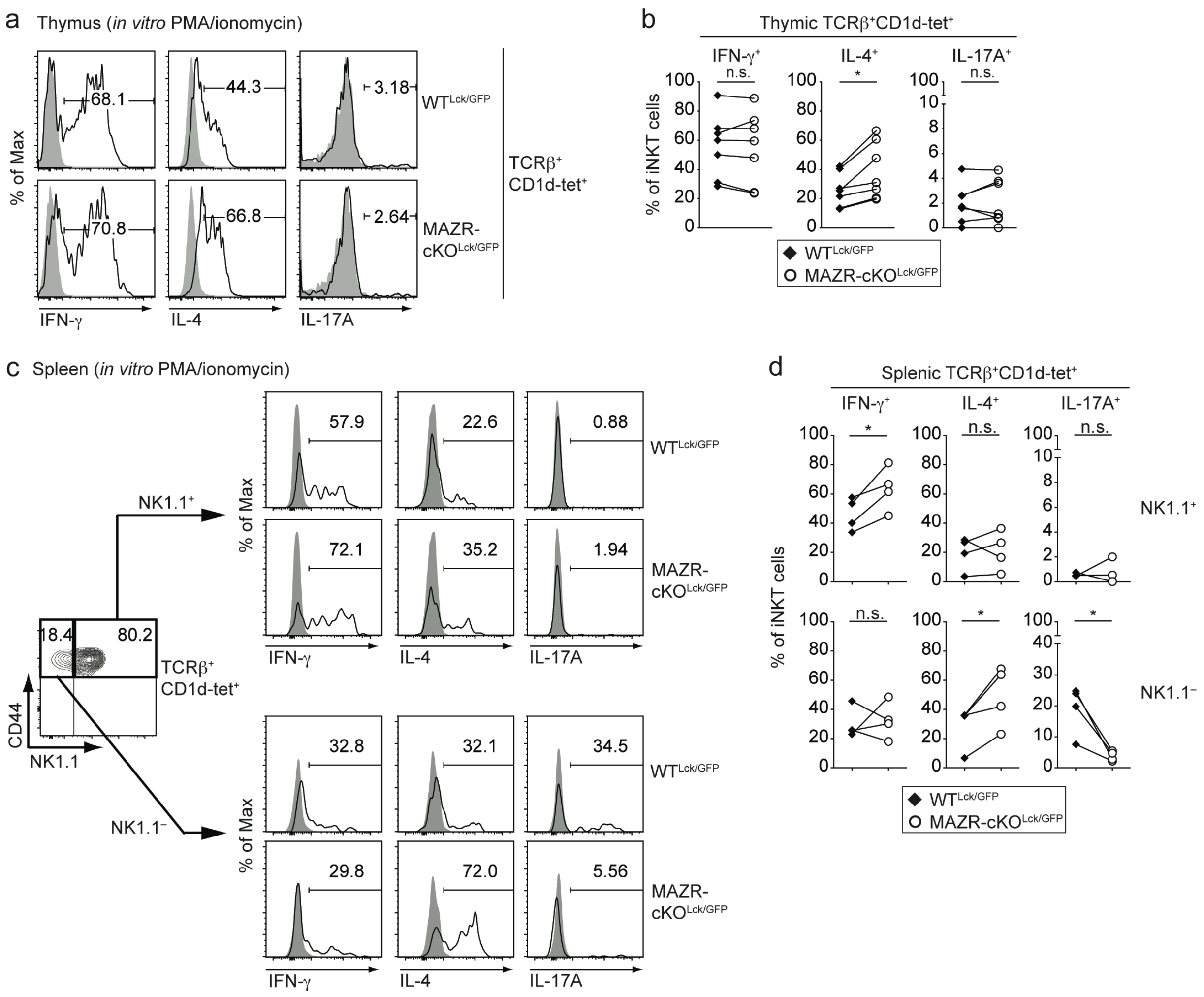

Fig. S5 Cytokine production in thymic as well as NK1.1$^+$ and NK1.1$^-$ splenic iNKT cells. **a** Histograms showing IFN-γ (left panel), IL-4 (middle panel) and IL-17A (right panel) expression in TCRβ$^+$CD1d-tet$^+$ thymocytes isolated from WT$^{Lck/GFP}$ (upper panel) and MAZR-cKO$^{Lck/GFP}$ (lower panel) mice. Data are representative of at least 10 mice analyzed in at least 5 independent experiments. **b** Diagrams showing the percentage of IFN-γ$^+$ (left panel), IL-4$^+$ (middle panel) and IL-17A$^+$ (right panel) splenic iNKT cells isolated from WT$^{Lck/GFP}$ and MAZR-cKO$^{Lck/GFP}$ mice. **c** Histograms showing IFN-γ (left panel), IL-4 (middle panel) and IL-17A (right panel) expression in TCRβ$^+$CD1d-tet$^+$NK1.1$^+$ splenocytes isolated from WT$^{Lck/GFP}$ (upper panel) and MAZR-cKO$^{Lck/GFP}$ (upper middle panel) mice as well as in TCRβ$^+$CD1d-tet$^+$NK1.1$^-$ splenocytes isolated from WT$^{Lck/GFP}$ (lower middle panel) and MAZR-cKO$^{Lck/GFP}$ (lower panel) mice. A representative gating strategy for the identification of NK1.1$^+$ and NK1.1$^-$ iNKT cell subsets is shown on the left side. Data are representative of 7-8 mice analyzed in 4 independent experiments. **d** Diagrams showing the percentage of IFN-γ$^+$ (left panel), IL-4$^+$ (middle panel) and IL-17A$^+$ (right panel) cells within splenic NK1.1$^+$ (upper panel) and NK1.1$^-$ (lower panel) iNKT cells isolated from WT$^{Lck/GFP}$ and MAZR-cKO$^{Lck/GFP}$ mice. **a,c** Thymocytes or splenocytes were stimulated with PMA and ionomycin for 4.5 hours in the presence of GolgiStop. Filled histograms show the expression level of each cytokine in unstimulated WT$^{Lck/GFP}$ TCRβ$^+$CD1d-tet$^+$ thymocytes or splenocytes. Numbers indicate the percentages of cells within the respective regions. **b,d** Each dot represents the average value of an individual experiment, in which 1-3 mice were analyzed per group. The lines indicate paired experiments. A paired two-tailed Student's *t*-test was performed for statistical analysis.

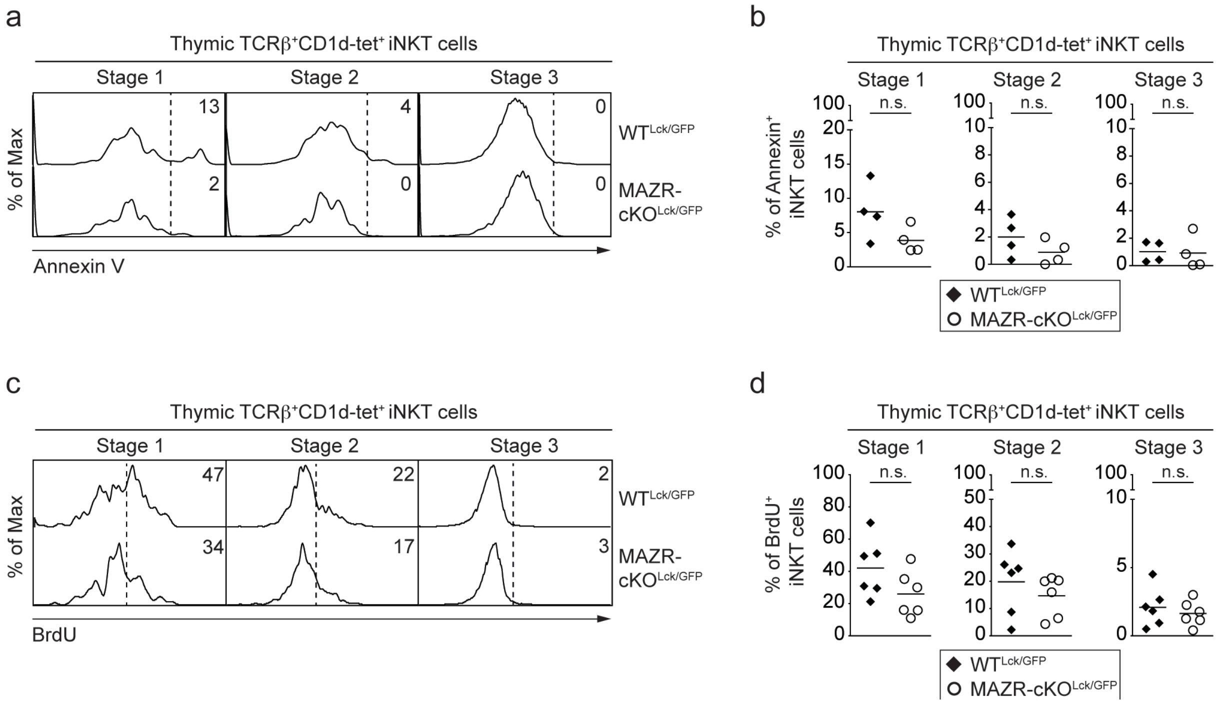

Fig. S6 Survival as well as proliferation of iNKT cells is intact in the absence of MAZR. **a** Histograms showing Annexin V expression in thymic stage 0 ($CD24^+CD44^-CD69^+$), stage 1 ($CD24^-CD44^-NK1.1^-$), stage 2 ($CD24^-CD44^+NK1.1^-$) and stage 3 ($CD24^-CD44^+NK1.1^+$) thymic iNKT cells isolated from $WT^{Lck/GFP}$ and MAZR-$cKO^{Lck/GFP}$ mice. Before Annexin V staining, iNKT cells were enriched by negatively depleting $CD8\alpha^+$ and $CD19^+$ cells. Numbers indicate the percentages of Annexin V^+ cells. Data are representative of 4 mice analyzed in 3 independent experiments. **b** Diagrams showing the percentage of Annexin V^+ cells within stage 1 (left panel), stage 2 (middle panel) and stage 3 (right panel) thymic iNKT cells isolated from $WT^{Lck/GFP}$ and MAZR-$cKO^{Lck/GFP}$ mice. Each dot represents one mouse. Horizontal bars indicate mean values. **c** Histograms showing BrdU incorporation in each developmental subset of thymic iNKT cells isolated from $WT^{Lck/GFP}$ and MAZR-$cKO^{Lck/GFP}$ mice. Mice were intraperitoneally injected 1 mg of BrdU, and were subsequently fed with BrdU-containing water (1 mg/ml). Forty-eight hours after injection mice were euthanized, and single cell suspensions of thymocytes were prepared. Subsequently, iNKT cells were enriched by negatively depleting $CD8\alpha^+$ and $CD19^+$ cells, and $BrdU^+$ cells within iNKT cell subsets were detected by flow cytometer. Numbers indicate the percentages of $BrdU^+$ cells. Data are representative of 6 mice analyzed in 4 independent experiments. **d** Diagrams showing the percentage of $BrdU^+$ cells within stage 1 (left panel), stage 2 (middle panel) and stage 3 (right panel) thymic iNKT cells isolated from $WT^{Lck/GFP}$ and MAZR-$cKO^{Lck/GFP}$ mice. Each dot represents one mouse. Horizontal bars indicate mean values.

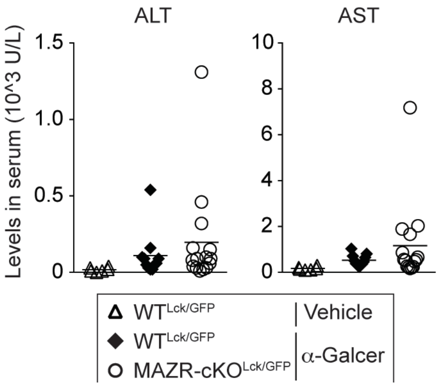

Fig. S7 Loss of MAZR led to no alteration in the degree of liver injury upon α-GalCer challenge. Diagrams showing alanine aminotransferase (ALT, left) and aspartate aminotransferase (AST, right) levels in serum isolated from α-GalCer-treated $WT^{Lck/GFP}$ and $MAZR\text{-}cKO^{Lck/GFP}$ mice. Serum samples were prepared twenty-four hours after α-GalCer administration. Each dot represents one mouse. Horizontal bars indicate mean values. A one-way ANOVA followed by Tukey's multiple comparison test was used for statistical analysis. No comparison between two groups reached a statistically significant level.

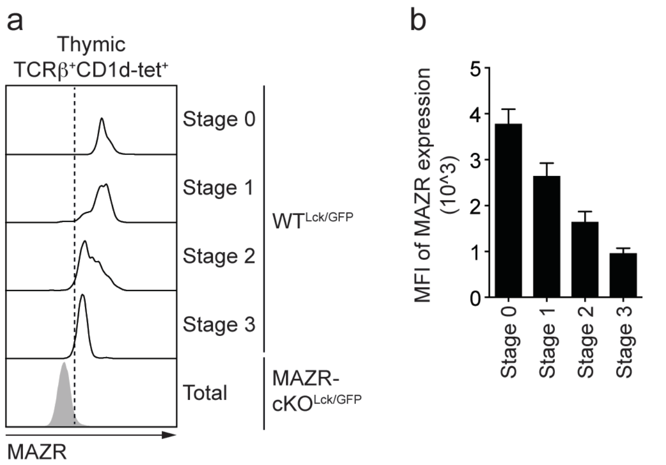

Fig. S8 Expression pattern of MAZR during thymic iNKT cell development. **a** Histograms showing MAZR expression in thymic stage 0 ($CD24^{+}CD44^{-}CD69^{+}$), stage 1 ($CD24^{-}CD44^{-}NK1.1^{-}$), stage 2 ($CD24^{-}CD44^{+}NK1.1^{-}$) and stage 3 ($CD24^{-}CD44^{+}NK1.1^{+}$) iNKT cells isolated from $WT^{Lck/GFP}$ mice. Total thymic iNKT cells isolated from MAZR-$cKO^{Lck/GFP}$ mice were provided as a negative control for MAZR staining. Dotted line indicates regions for $MAZR^{+}$ population. **b** Diagrams showing mean fluorescent intensity (MFI) of MAZR expression in each developmental subset of thymic iNKT cells isolated from $WT^{Lck/GFP}$ mice. Data are presented as mean values with standard errors. **a,b** Data are representative (**a**) or summary (**b**) of 5 mice analyzed in 5 independent experiments.

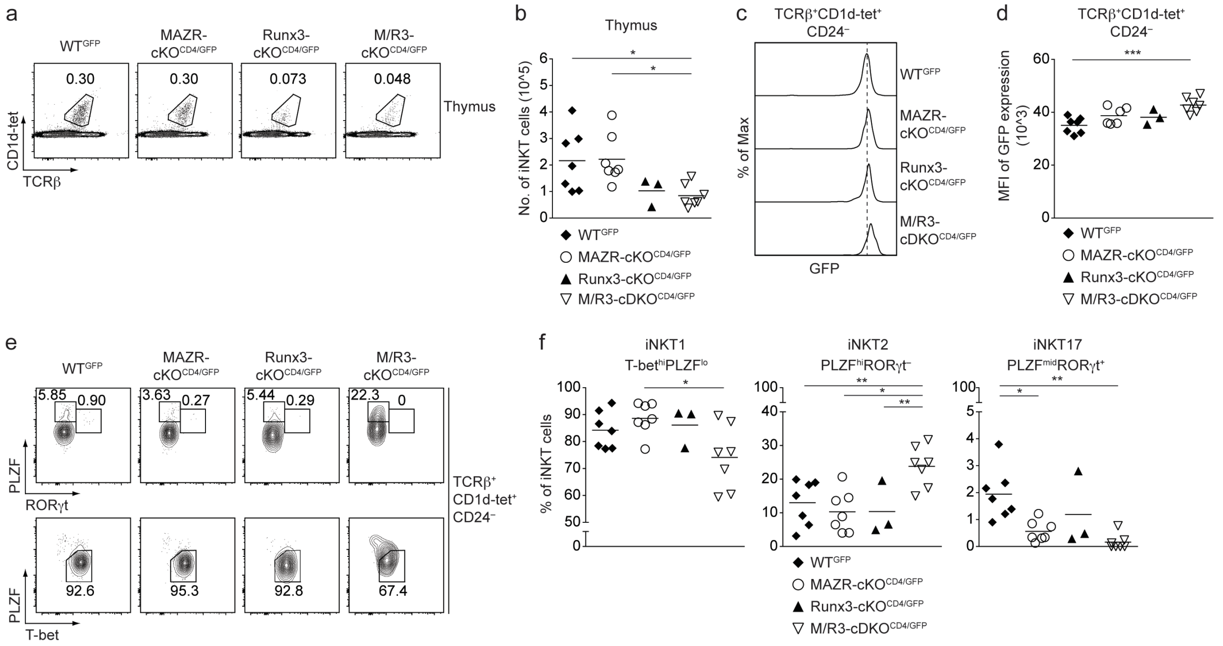

Fig. S9 MAZR and Runx3 synergistically control thymic iNKT cell development. **a** Flow cytometry analysis showing CD1d-tet and TCRβ expression on thymocytes isolated from *Mazr*$^{f/f}$*Runx3*$^{f/f}$*Thpok*$^{+/GFP}$, *Mazr*$^{f/f}$*Runx3*$^{+/+}$*Thpok*$^{+/GFP}$*Cd4*-Cre, *Mazr*$^{+/+}$*Runx3*$^{f/f}$*Thpok*$^{+/GFP}$*Cd4*-Cre and *Mazr*$^{f/f}$*Runx3*$^{f/f}$*Thpok*$^{+/GFP}$*Cd4*-Cre mice (hereafter referred to as WT^{GFP}, MAZR-cKO$^{CD4/GFP}$, Runx3-cKO$^{CD4/GFP}$ and M/R3-cKO$^{CD4/GFP}$ mice). Numbers indicate the percentages of TCRβ^{+}CD1d-tet^{+} cells. **b** Diagrams showing the total numbers of thymic iNKT cells isolated from WT^{GFP}, MAZR-cKO$^{CD4/GFP}$, Runx3-cKO$^{CD4/GFP}$ and M/R3-cKO$^{CD4/GFP}$ mice. **c** Histograms showing GFP expression (i.e. *Thpok* expression) in TCRβ^{+}CD1d-tet^{+}CD24^{-} thymocytes isolated from WT^{GFP}, MAZR-cKO$^{CD4/GFP}$, Runx3-cKO$^{CD4/GFP}$ and M/R3-cKO$^{CD4/GFP}$ mice. Dotted vertical lines indicate the peaks of GFP expression in WT^{GFP} cells. **d** Diagrams showing the mean fluorescence intensity (MFI) of GFP expression in TCRβ^{+}CD1d-tet^{+}CD24^{-} thymocytes isolated from WT^{GFP}, MAZR-cKO$^{CD4/GFP}$, Runx3-cKO$^{CD4/GFP}$ and M/R3-cKO$^{CD4/GFP}$ mice. **e** Flow cytometry analysis showing PLZF and Rorγt expression (upper panel) and PLZF and T-bet expression (lower panel) in TCRβ^{+}CD1d-tet^{+}CD24^{-} thymocytes isolated from WT^{GFP}, MAZR-cKO$^{CD4/GFP}$, Runx3-cKO$^{CD4/GFP}$ and M/R3-cKO$^{CD4/GFP}$ mice. Numbers indicate the percentages of cells within the respective regions. **f** Diagrams showing the percentage of thymic iNKT1 (T-bet^{hi}PLZF^{lo}: left panel), iNKT2 (PLZF^{hi}RORγt^{-}: middle panel) and iNKT17 (PLZF^{mid}RORγt^{+}: right panel) cells in WT^{GFP}, MAZR-cKO$^{CD4/GFP}$, Runx3-cKO$^{CD4/GFP}$ and M/R3-cKO$^{CD4/GFP}$ mice. **a,c,e** Data are representative of 3-7 mice analyzed in 3 independent experiments. **b,d,f** Each dot represents one mouse. Horizontal bars indicate mean values. A one-way ANOVA analysis followed by Tukey's multiple-comparison test was performed for statistical analysis. Differences that did not reach a statistically significant level (i.e. $p \geq 0.05$) are not indicated.

Of note: For the experiments shown in this figure, MAZR was deleted using Cd4-Cre. The percentage of iNKT17 cells was reduced in MAZR-cKO$^{CD4/GFP}$ *mice, as observed in MAZR-cKO*$^{Lck/GFP}$ *mice (Fig. S2). However, the degree of the increase in ThPOK expression was smaller in MAZR-cKO*$^{CD4/GFP}$ *iNKT cells (approx. 1.1-fold), compared to in MAZR-cKO*$^{Lck/GFP}$ *cells (approx. 1.4-fold, see also Fig. 2c and 2d). In addition, there was no increase in the proportion of iNKT2 cells MAZR-cKO*$^{CD4/GFP}$ *mice, although this was observed in MAZR-cKO*$^{Lck/GFP}$ *mice (Fig. S2). This indicates some differences in iNKT cell subset phenotypes between MAZR-cKO*$^{Lck/GFP}$ *and MAZR-cKO*$^{CD4/GFP}$ *mice. Some phenotypic differences between these strains of mice have been also observed for conventional T cell development, including the regulation of ThPOK expression, and this was linked to differences in the kinetic of Mazr deletion (Sakaguchi et al., J. Immunol. 2015; PMID: 26254341). Therefore, the differences in iNKT cell development between the two MAZR-deficient mouse strains might be most likely due to a difference in the timing of Mazr deletion (owing to the differential usage of Cre-lines), and as a consequence of different residual amounts of MAZR protein being expressed during the course of iNKT cell development.*

Antigen	Clone or Catalog number (#)	Company
CD24	M1/69	Thermo Fisher Scientific
CD27	LG3A10	BD Biosciences
CD4	RM4-5	Thermo Fisher Scientific
CD44	IM7	Biolegend
CD45.1	A20	Biolegend
CD45.2	104	Thermo Fisher Scientific
CD69	H1.2F3	Biolegend
CD8α	53-6.7	Thermo Fisher Scientific
c-Myc	D84C12	Cell Signaling Technology
Egr2	erongr2	Thermo Fisher Scientific
GATA-3	TWAJ	Thermo Fisher Scientific
IFN-γ	XMG1.2	BD Biosciences
IL-17A	eBio17B7, TC11-18H10.1	Thermo Fisher Scientific, Biolegend
IL-17RB	MUNC33	Thermo Fisher Scientific
IL-4	11B11	BD Biosciences
MAZR/PATZ1	D-5	Santa Cruz Biotechnology
Mouse IgG_1	RMG1-1	Biolegend
NK1.1	PK136	Biolegend
PLZF	9E12, R17-809	Biolegend, BD Biosciences
Rabbit IgG	#8885	Cell Signaling Technology
RORγt	Q31-378	BD Biosciences
T-bet	4B10	Biolegend
TCRβ	H57-597	Thermo Fisher Scientific

Table S1: Antibodies used in this study.
